# A Multiplex Snapback Primer System for the Enrichment and Detection of *JAK2* V617F and *MPL* W515L/K Mutations in Philadelphia-Negative Myeloproliferative Neoplasms

**DOI:** 10.1155/2014/458457

**Published:** 2014-03-05

**Authors:** Zhiyuan Wu, Yunqing Zhang, Xinju Zhang, Xiao Xu, Zhihua Kang, Shibao Li, Chen Zhang, Bing Su, Ming Guan

**Affiliations:** ^1^Central Laboratory, Huashan Hospital, Shanghai Medical College, Fudan University, Shanghai, China; ^2^Department of Laboratory Medicine, Huashan Hospital North, Shanghai Medical College, Fudan University, Shanghai, China; ^3^Department of Dermatology, The Third Affiliated Hospital of Sun Yat-sen University, Guangzhou, China; ^4^Department of Laboratory Medicine, Huashan Hospital, Shanghai Medical College, Fudan University, Shanghai, China; ^5^Department of Cancer Genetics, Roswell Park Cancer Institute, Buffalo, NY, USA

## Abstract

A multiplex snapback primer system was developed for the simultaneous detection of *JAK2* V617F and *MPL* W515L/K mutations in Philadelphia chromosome- (Ph-) negative myeloproliferative neoplasms (MPNs). The multiplex system comprises two snapback versus limiting primer sets for *JAK2* and *MPL* mutation enrichment and detection, respectively. Linear-After exponential (LATE) PCR strategy was employed for the primer design to maximize the amplification efficiency of the system. Low ionic strength buffer and rapid PCR protocol allowed for selective amplification of the mutant alleles. Amplification products were analyzed by melting curve analysis for mutation identification. The multiplex system archived 0.1% mutation load sensitivity and <5% coefficient of variation inter-/intra-assay reproducibility. 120 clinical samples were tested by the multiplex snapback primer assay, and verified with amplification refractory system (ARMS), quantitative PCR (qPCR) and Sanger sequencing method. The multiplex system, with a favored versatility, provided the molecular diagnosis of Ph-negative MPNs with a suitable implement and simplified the genetic test process.

## 1. Introduction

The classical myeloproliferative noeplasms (MPNs), according to the definition of World Health Organization (WHO) classification criteria revised in 2008, encompass chronic myelogenous leukemia (CML), polycythemia vera (PV), essential thrombocythemia (ET), and primary myelofibrosis (PMF) [1]. In 2005, the discovery that somatic mutation in *JAK2* exon 14 (*JAK2* V617F) is presented in almost all the PV and half of the ET and PMF patients totally modified our understanding of these Philadelphia chromosome- (Ph-) negative MPNs [2–5]. Soon afterwards, somatic mutations in *MPL* exon 10 (*MPL* W515L/*MPL* W515K) were also identified in around 10% of the *JAK2* V617F mutation-negative ET and PMF patients [6, 7]. These *JAK2* and *MPL* mutations are also considered as the driver molecular alternation of the disease because of the resulting constitutive activation of JAK-STAT pathway. Consequently, these mutations are now incorporated into the diagnostic criteria of the Ph-negative MPNs [8].

The prevalent tools for the identification of these mutations are mostly based on the common techniques such as sequencing, allele specific PCR (also known as the amplification refractory mutation system, ARMS), and quantitative PCR (qPCR). However, there are also several major defects in these methods. For instance, sequencing and allele specific PCR are both labor-intensive and time consuming due to the manual post-PCR procedures, which lead to the unfavorable turnaround time and latent risk of DNA contamination. Although the allele specific qPCR is an efficient closed-tube procedure for mutation identification, this fluorescent based method still costs false positive results [9]. Moreover, MPNs are characterized by the independent origins of genetically distinct clones [10], contributing to the relatively low mutation load of these JAK-STAT pathway mutations in a large proportion of patients, especially *MPL* mutations in ET and PMF. Unfortunately, Sanger sequencing, the current gold standard for mutation identification, is not able to detect these low-abundance mutations (mutation load < 20%) [11]. Besides, the additional rare mutation in the template can even introduce false negative test result during the allele specific PCR assay [12]. Thus, the development of a highly sensitive, easy to operate, and efficient detecting method for the mutations in Ph-negative MPNs is urgently needed for the accurate diagnosis and prognosis of disease, as well as the minimal disease monitoring.

Inspired by the scorpion probe, Zhou et al. [13] developed the snapback primer assay system with saturating DNA dye for the enrichment and detection of the rare-allele mutation. Characterized by an extremely high sensitivity detection of mutations, the snapback primer system can identify almost all the mutations flanked by the snapback probe during the melting curve analysis.

In this study, we employed the snapback primer concept and constructed a multiplex snapback primer system for simultaneous detection of trace-amount *JAK2* V617F and *MPL* W515L/K mutations in the classical Ph-negative MPNs patients.

## 2. Materials and Methods

### 2.1. DNA Extraction from Cell Lines

*JAK2* V617F homozygous mutant human erythroleukemia (HEL) cell line and *JAK2*/*MPL* homozygous wild-type multiple myeloma (RPMI 8226) cell line were purchased from the cell bank of type culture collection of Chinese Academy of Sciences. The HEL cell line was used as *JAK2* V617F mutation-positive control, while the RPMI8226 cell line was used as *JAK2* V617F and *MPL* W515L/K mutation-negative control. Genomic DNA from the cells was extracted with QiaAmp DNA Blood Mini Kit (Qiagen, Valencia, CA) according to the manufacturer's instruction to achieve the final concentration of 20 ng/*μ*L.

### 2.2. Construction of Artificial Plasmids

Artificial plasmids with the *MPL* W515L and *MPL* W515K were constructed with pMD19-T simple vector utilizing the overlap mutagenesis technology [14]. The insert fragment flanked NG_007525.1:g.16123-16775 and all the wild-type and mutant insertions were verified by Sanger sequencing. After the validation of a SYBR Green based qPCR assay, all the artificial plasmids were finally diluted to the identical *MPL* amplicon copy number as in the 20 ng/*μ*L genomic DNA from RPMI8226 cells with deionized distilled water (ddH_2_O) (3 × 10*E*-4 ng/*μ*L).

### 2.3. Patient Samples

The peripheral blood samples were obtained from 50 PV, 50 ET, and 20 PMF patients in the Department of Hematology in Huashan Hospital of Fudan University. All these Ph-negative patients were diagnosed according to [8]. Written informed consents were received from all the participants. DNA was extracted from the blood samples collected in ethylenediaminetetraacetic acid anticoagulant with QIAamp DNA Blood Mini Kit and diluted with ddH_2_O to a final concentration of 15–25 ng/*μ*L. In compliance with Helsinki Declaration of 1975 as revised in 1996, this study was approved by the Institutional Review Board of Huashan Hospital.

### 2.4. Enrichment of *JAK2* V617F and *MPL* W515L/K Mutation with Multiplex Snapback Primer System

The multiplex snapback primer system was developed on a Rotor-Gene Q real-time PCR platform (Qiagen). The primer sequences used for PCR are listed in Table 1. The snapback primers comprised two parts, the snapback probe at the 5′-end and the conventional annealing primer at the 3′-end. Thus, during PCR annealing, the 3′-primer will anneal to the template for the subsequent extension and the 5′-snapback probe will combine with the target sequence of PCR amplicon, which will finally form the stem-loop secondary structure for rare-allele enrichment and detection. To eliminate the unfavorable extension of the snapback probe during PCR, the 5′-terminal of the snapback primers was blocked with 2 nucleotides that mismatched the target sequence. The limiting primers were developed with the concept of linear-after-the-exponential (LATE) PCR [15]. In other words, the melting temperature (*T*_*m*_) of the limiting primer was 4-5°C higher than its paired snapback primer. Therefore, both the excessive snapback primer and the limiting primer can achieve the same annealing activity during the asymmetric PCR procedure.

We performed asymmetric PCR with the TaKaRa Ex Taq Hot Start Version Kit (TaKaRa BIO, Shiga, Japan) in a 20 *μ*L of reaction volume. The master mix contained 1U TaKaRa Ex Taq HS, 2 *μ*L 10 × Ex Taq buffer (Mg^2+^ free), 0.5 mM MgCl_2_, 0.25 *μ*M *JAK2* V617F snapback primer, 0.02 *μ*M *JAK2* V617F limiting primer, 0.25 *μ*M *MPL* W515L/K snapback primer, 0.02 *μ*M *MPL* W515L/K limiting primer, 1.5 *μ*M SYTO-9 DNA dye (Invitrorgen, Carlsbad, CA), and 15–25 ng DNA template. The PCR was performed on a Rotor-Gene Q real-time platform. The PCR protocol included an initial denaturation step (95°C for 10 min) followed by 70 cycles of 95°C for 1 sec, 58°C for 1 sec, and 72°C for 1 sec. The products were then heated for denaturation at 98°C for 2 min, followed by cooling down to 40°C for 2 min to facilitate the hybridization of snapback probe, and then melted at a ramping rate of 0.5°C/sec from 50°C to 99°C. High resolution melting- (HRM-) curve analysis was performed with the Rotor-Gene Q 1.7 software. For each assay, the positive and negative controls for both *JAK2* V617F and *MPL* W515L/K mutations were always included.

### 2.5. Analytical Sensitivity and Reproducibility of Multiplex Snapback Primer System

Serial dilution of homozygous HEL cell line DNA was performed with the wild-type RPMI 8226 cell line DNA to prepare the standards of 10%, 1%, 0.1%, and 0.01% HEL cell DNA load. Meanwhile the *MPL* W515L and *MPL* W515K mutation stands were also made up with serial dilution of the *MPL* artificial plasmids and RPMI 8226 cell line DNA. The analytical sensitivity for *JAK2* V617F and *MPL* W515L/K mutation of this snapback system was then evaluated by testing these standards. The reproducibility of this system was confirmed with 20 intra-assay duplications and a 20-day interassay duplication by testing the standards with the minimum detectable mutation load.

### 2.6. Detection of *JAK2* V617F and *MPL* W515L/K Mutations by ARMS Method

The ARMS PCR testing for *JAK2* V617F mutation [4] and *MPL* W515L/K mutations [16] was performed as described. The assay for *JAK2* V617F identification utilized a pair of outer primers as well as wild-type/mutant specific primers to amplify the wild-type or mutant fragment with an internal amplification control sequence in a single reaction. Detection of *MPL* W515L/K mutations was carried out in a single PCR reaction with 2 outer primers designed to amplify the internal amplification control flanking the mutation site. The 2 specific primers for either the wild-type or the mutant sequence were employed to distinguish the wild-type allele from the mutant one by fragment size. A similar assay was also developed to detect *MPL* W515K mutation with the other mutation specific primer. All the primers used in the ARMS method are listed in Table 1. Amplifications were performed for 35 cycles with the HotStarTaq Master Mix Kit (Qiagen).

### 2.7. Detection of *JAK2* V617F and *MPL* W515L/K Mutations by TaqMan Probe qPCR

Two qPCR system based on the TaqMan hydrolysis probe was performed to detection of *JAK2* V617F [17] and *MPL* W515L/K mutations [18] as described, respectively. The primer and TaqMan probe sequences and fluorescent labels were listed in Table 1. The *JAK2* V617F detection system comprises a single set of primers and two probes that differed only at the position of V617F mutant nucleotide substitution and fluorescent labelling, so as to discriminate the mutation from the wild-type sequence in a single qPCR reaction. The *MPL* W515L/K mutation detecting system consisted of the same amplification primers but the distinct TaqMan probe that is specifically designed for the wild-type W515L and W515K mutant template. The amplification and fluorescent signal acquiring were performed for 40 cycles with the Quantitect Probe qPCR kit (Qiagen) on a LightCycler-480 II qPCR platform (Roche, Basel, Switzerland) according to the manufacturer's instructions. The qPCR assay data were acquired and analyzed with the LC-480 software Version 1.5.

### 2.8. DNA Sequencing and T-A Cloning for *JAK2* V617F and *MPL* W515L/K Mutations

To verify the result of snapback primer assay, we amplified 10 *JAK2* V617F-positive samples, all the 5 *MPL* W515L/K-positive samples (3 W515L and 2 W515K), and 10 mutant allele-free samples with the sequencing primers listed in Table 1. Afterwards, all the products were subjected to bidirectional Sanger sequencing using these sequencing primers. The outcome sequences of each assay for the *JAK2* V617F and *MPL* W515L/K detection were aligned with the reference sequence of *JAK2* (NG_009904.1) and *MPL* (NG_007525.1), respectively. In consideration of the limited rare-allele detecting sensitivity, all the mutation-negative amplicons identified by direct sequencing were subjected to T-A cloning in order to produce the fragments with monoclonal allele. These amplicons (377 bp for *JAK2* and 184 bp for *MPL*) were separated with 1.5% agarose electrophoresis and purified using the Qiaquick gel purification kit (Qiagen). For each mutation, the purified extracts were mixed with the pMD19-T simple vector master mix (Takara BIO) according to the manufacturer's instruction and ligated at 16°C for 2 hours. The vector plasmids with cloned insert were transformed into DH5*α* competent *E. coli* cells by 42°C heat shock for 15 seconds and iced chilling. Transformed *E. coli* cells were multiplied at 37°C in the Luria-Bertani (LB) broth for 1 hour and then spread onto the IPTG/x-GAL (Invitrogen) coated ampicillin-LB agar dishes. After 37°C incubation for 16 hours, the white clones were picked up and again enriched in the ampicillin-LB broth at 37°C overnight. Plasmid DNA of each single clone was extracted with Plasmid Mini Kit (Qiagen) according to the manufacturer's instruction and subjected to sequencing with the correspondent sequencing primers. For each dish, a maximum of 100 clones were sequenced, till the isolate with mutant allele was identified. All the Sanger sequencing assays were performed on an Applied Biosystems PRISM 3130 genetic analyzer in the Invitrogen Laboratory of Technical Services (Shanghai).

## 3. Results

### 3.1. Enrichment and Detection of *JAK2* V617F Mutation and *MPL* W515L/K Mutations by Multiplex Snapback Primer System

For each snapback primer set in the multiplex system, the asymmetric PCR produced two kinds of amplicon. The snapback primer and the limiting primer first generated double-strand DNA fragment. After the depletion of limiting primer, the excess snapback primer produced single strand amplicon with snapback probe tail in a linear amplification manner. The snapback probe was complementary to the target sequence in the single-strand amplicon, which led to the formation of the stem-loop hairpin of the snapback primer's own extension product. Since the snapback probe was designed totally complement to the wild-type allele, the wild-type hairpin possessed a higher thermal stability than the mutant one. Under the circumstance of low Mg^2+^ concentration and rapid PCR protocol (momentary denaturation/annealing/extension duration), the mutant hairpin could be more easily denatured for further template amplification. After dozens of PCR cycles, the mutant allele was selectively amplified.

**Figure 1:**
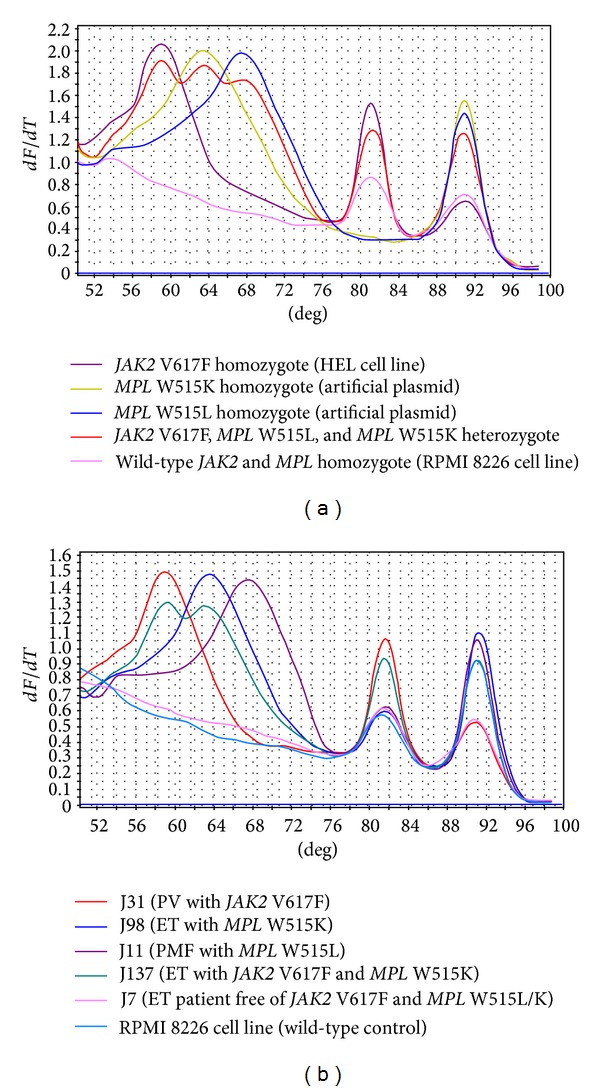
Detection of *JAK2* V617F, *MPL* W515L, and *MPL* W515K mutations with a multiplex snapback primer system. Negative first-derivative (*dF/dT*) plot of melting curve consists of two melting regions. The stem-loop hairpin melting region for mutation discrimination (55–75°C) and the double-strand amplicon for DNA template amplification control (75–95°C). (a) Samples with the *JAK2* V617F mutation (purple) showed a melting peak at 59.3°C, while the melting peaks at 63.2°C and 67.6°C indicated the presence of *MPL* W515K (ginger) and *MPL* W515L (blue) mutations, respectively. The amplification controls of *JAK2* and *MPL* were presented by the melting peak at 81.1°C and 91.2°C, respectively. The mixture of three mutant allele-positive standards (red) generated all the three mutation melting peaks. In wild-type control sample (pink), only the double-strand control was amplified. (b) The amplicon melting curves of DNA from patients with *JAK2* V617F, *MPL* W515L, *MPL* W515K, and concurrent *JAK2* V617F and *MPL* W515K mutation.

In the presence of saturating fluorescent DNA dye, both the stem-loop hairpins and double-strand DNA amplicons could be distinguished as the melting peaks by plotting the negative derivative of fluorescence versus the melting temperature. Amplicon with different alleles resulted in different melting transitions; thus the templates with different mutations could be easily discriminated from each other. In the melting curve analysis, the double-strand DNA amplicon of each sample was utilized as the amplification control. The stem-loop hairpin with *JAK2* V617F, *MPL* W515K, and *MPL* W515L allele was observed at 59.3°C, 63.2°C, and 67.6°C, respectively. Meanwhile, the double strand amplicons of *JAK2* and *MPL* gene were presented at 81.1°C and 91.2°C (Figure 1(a)).

### 3.2. Analytical Sensitivity and Reproducibility of Multiplex Snapback Primer System

**Figure 2:**
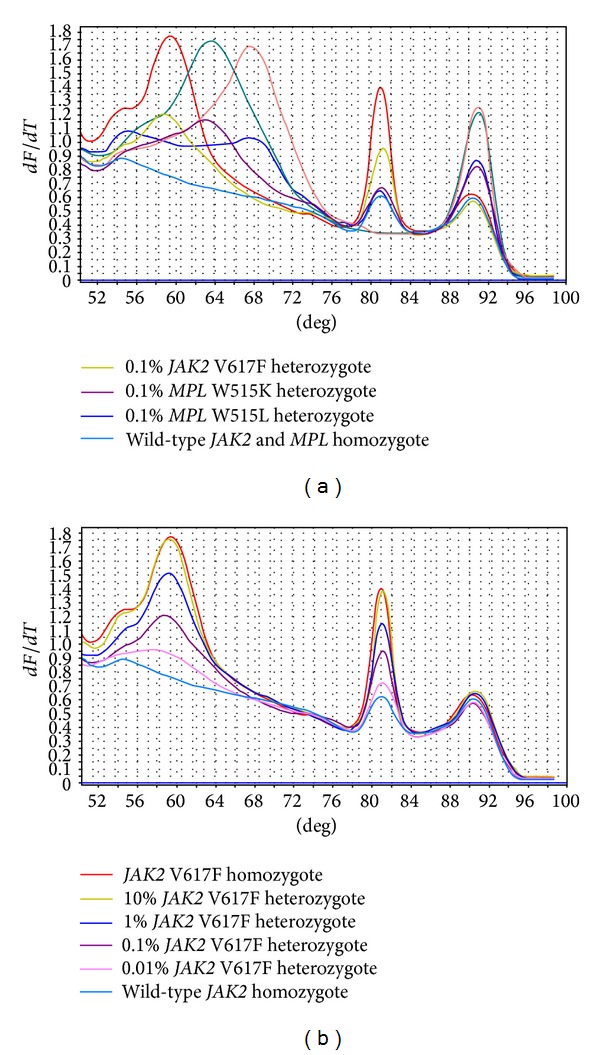
Analytical sensitivity of the multiplex snapback primer system for mutant allele discrimination. (a) Serial diluted standards of 0.1% load *MPL* W515L (blue), *MPL* W515K (purple), and *JAK2* V617F (ginger) specifically generated the corresponding melting peaks, which could be easily discriminated from the wild-type control (lake blue). (b) Serial *JAK2* V617F dilution of 0.01% (pink), 0.1% (purple), 1% (blue), and 10% (ginger) mutation load. After the robust mutation enrichment, snapback primer system generated a melting curve with 0.1% mutation load that can be distinguished from the wild-type control.

The sensitivity of this multiplex system for each mutant allele was evaluated with the corresponding mutation standards (0.01%, 0.1%, 1%, and 10% HEL cell DNA load). Therefore, up to 0.1% (10 copies/*μ*L) *JAK2* V617F and *MPL* W515L/K mutation can be discriminated from the wild-type allele after the melting curve analysis (Figure 2).

The Intra- and interassay precision of the system was studied by the coefficient of variation (CV) of melting peaks for each mutation allele. At a *JAK2* V617F mutation load of 0.1%, the intra- and interassay CVs of the system were 2.13% and 3.22%, respectively. Meanwhile, the system could also detect 0.1% load *MPL* W515L/K mutation with the intra- and interassay CVs of 1.22% and 1.85%, respectively. These results demonstrated the high sensitivity of the multiplex snapback primer system.

### 3.3. Detection of *JAK2* V617F and *MPL* W515L/K Mutations in Ph-Negative MPNs Patients by Multiplex Snapback Primer System

The snapback system identified 47 out of 50 PV (94%), 23 out of 50 ET (46%), and 8 out of 20 PMF (40%) patients harboring the* JAK2* V617F mutation. This system also detected 2 *MPL* W515L (2.5%), 1 *MPL* W515K (1.3%) mutation in the ET patients and 1 *MPL* W515L (5%), 1 *MPL* W515K (5%) in the PMF patients. The melting curve plots relating to the MPNs patient were illustrated in Figure 1(b). No *MPL* W515L or K mutation was found in the PV patients. No patient harbored both the *MPL* W515L and *MPL* W515K mutation. It is worth noting that one of the ET sample turned out to be *JAK2* V617F and *MPL* W515K mutation-positive, which is a rare clinical phenomenon.

### 3.4. Detection of *JAK2* V617F and *MPL* W515L/K Mutations in Ph1-Negative MPNs Patients by ARMS, TaqMan Probe qPCR, and Sanger Sequencing

**Figure 3:**
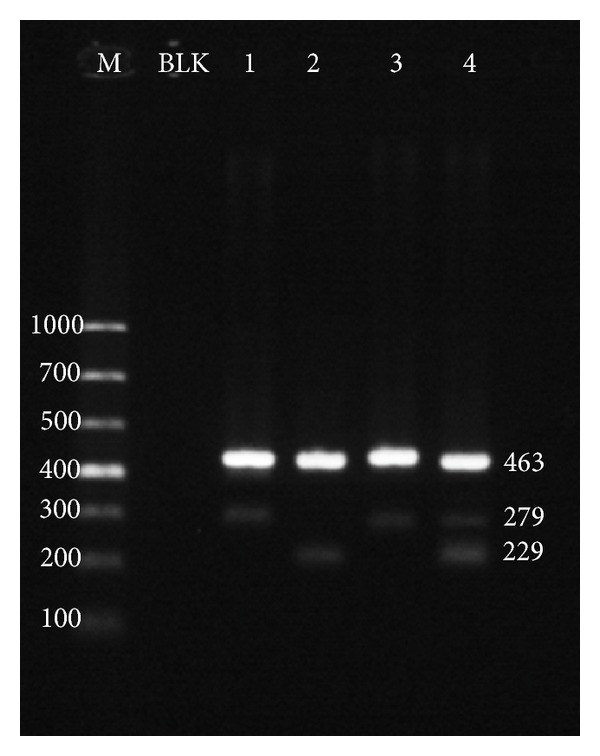
ARMS for *JAK2* V617F mutation. Amplified DNA products from the ARMS assay were subjected to 2% agarose electrophoresis for mutation identification. The 469 bp product served as the amplification control. Bands of 229 bp suggested the presence of the wild-type allele, while the mutant allele was indicated by the bands of 279 bp. M: 1,000 bp DNA ladder; BLK: no template amplification control; 1: HEL cell line DNA; 2: RPMI 8226 cell line DNA; 3: patient sample identified as *JAK2* V617F-homozyous positive; 4: patient sample with heterozygous *JAK2* V617F mutation.

The *JAK2* V617F ARMS assay utilized a pair of outer primers to produce a 463 bp amplification control flanking the mutation region. The 229 bp product of wild-type specific primer and reverse outer primer indicate the presence of wild-type allele in the sample. The mutation-specific primer and forward outer primer will generate a 279 bp fragment when the *JAK2* V617F mutant allele is present (Figure 3).

**Figure 4:**
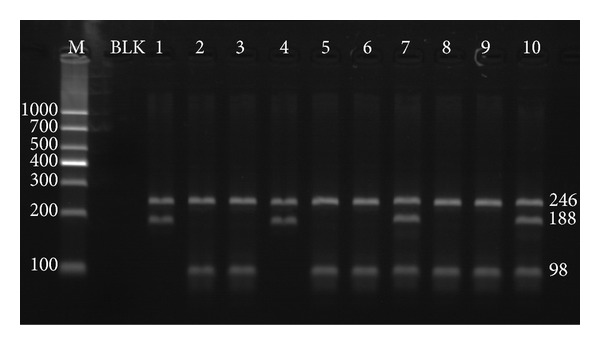
ARMS for *MPL* W515L and *MPL* W515K mutation. The *MPL* W515L/K ARMS products after 2% agarose electrophoresis. The 246 bp band represented the amplification control. Bands of 98 bp were the wild-type allele product. Presence of the 188 bp amplicon suggested the mutant allele in DNA sample. *MPL* W515L ARMS system was in tracks of 1, 3, 5, 7, and 9; *MPL* W515K ARMS system was in tracks of 2, 4, 6, 8, and 10. M: 1,000 bp DNA ladder; BLK: no template amplification control; 1, 2: artificial plasmid with *MPL* W515L allele; 3, 4: artificial plasmid with *MPL* W515K allele; 5, 6: RPMI 8226 cell line DNA; 7, 8: patient sample with heterozygous *MPL* W515L mutation; 9, 10: patient sample with heterozygous *MPL* W515K mutation.

The ARMS for *MPL* W515L/K mutation contained two assays for the W515L and W515K mutation, respectively. In each assay, the 246 bp band represents the amplification control. The 188 bp and 98 bp amplicons indicated the existence of mutant and wild-type allele, respectively (Figure 4).

In the 120 peripheral blood DNA from MPNs patients, the ARMS results were identical to those from the snapback primer assay, suggesting that the later one had a favorable analytical sensitivity, which is analogous to the conventional electrophoresis based method.

**Figure 5:**
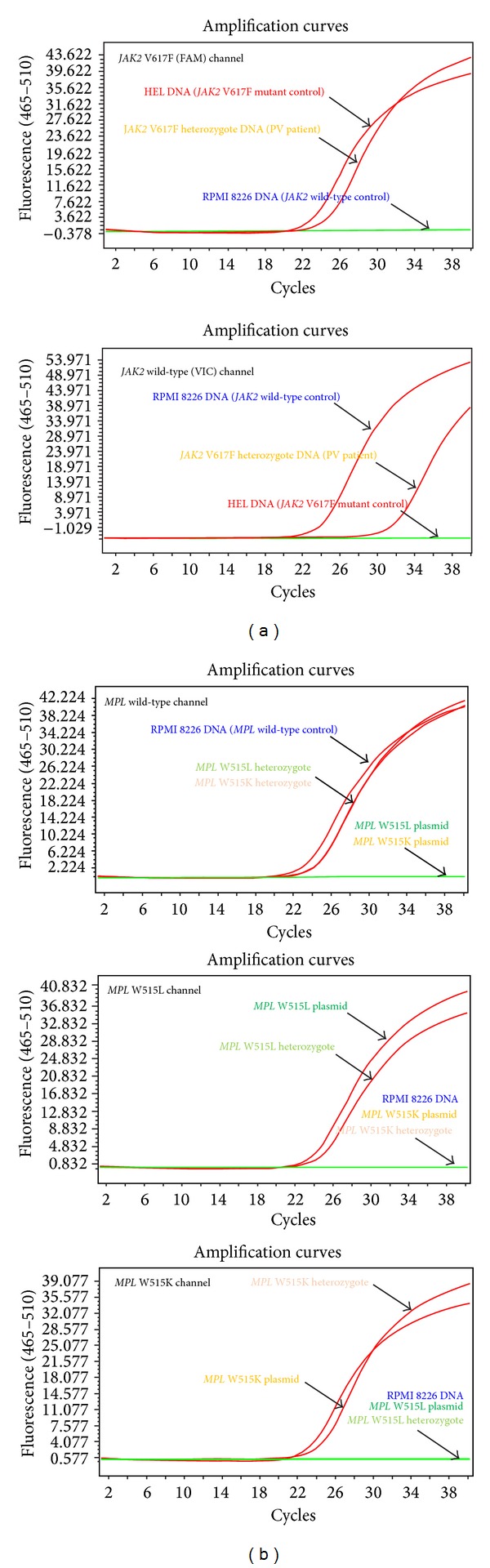
TaqMan probe qPCR assay for *JAK2* V617F and *MPL* W515L/K mutation. (a) TaqMan system for *JAK2* V617F detection. A sigmoidal shaped amplification curve in the fluorescent signal versus cycle number plot of FAM fluorescence channel (465 nm–510 nm) indicated the presence of *JAK2* V617F mutant allele in the DNA sample, while the amplification curve in VIC channel (540 nm–580 nm) represents the wild-type* JAK2* copies in the sample. *JAK2* V617F and *JAK2* wild-type homozygotes specifically produced the amplification curve in the FAM and VIC channel, respectively. The amplification of mutation heterozygote could be observed both in the FAM and VIC channel. (b) TaqMan system for *MPL* W515L/K mutations. The system was consisted of three allele-specific channels. The amplification curve of homozygous mutant and wild-type samples was only generated in the corresponding channel. The amplification curves of heterozygote with *MPL* W515L or *MPL* W515K mutation would be observed in both the wild-type channel and the mutant allele channel.

Results from the TaqMan assay for *JAK2* V617F mutation can be interpreted by observing the amplification plot in the FAM (mutant) and VIC (wild-type) fluorescence channel, while results of the *MPL* W515L/K mutation detecting system should be analyzed with the amplification curves obtained from three reaction wells (wild-type, W515L, and W515K). After the qPCR procedure, a sigmoidal shaped amplification curve in the fluorescent signal versus cycle number plot with a threshold cycle (*C*_*t*_) < 35 indicated the explicit existence of the corresponding allele, while the sample with a *C*_*t*_ > 38 amplification curve was considered free from the particular allele (Figure 5).

**Figure 6:**
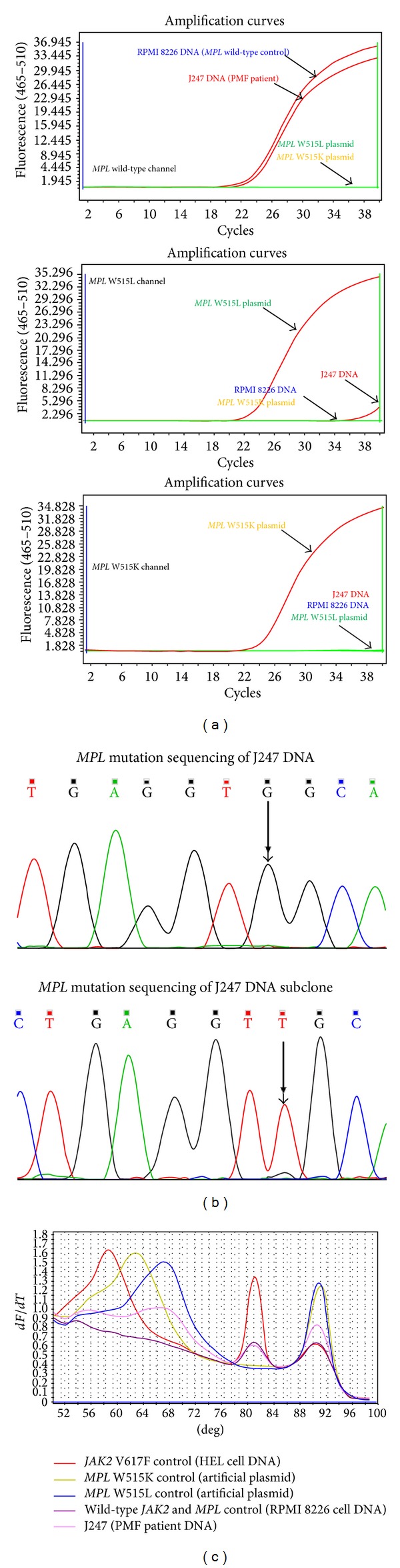
Snapback primer assay identifying a patient DNA with *MPL* W515L mutation. (a) Peripheral blood DNA from a patient with PMF (J247) was detected as weak* MPL* W515L mutation positive (*C*_*t*_ = 38.623). (b) Sanger sequencing of *MPL* W515L/K mutation after amplicon T-A cloning identified purified *MPL* W515L mutation clone. (c) Snapback primer assay discriminated the *MPL* W515L mutation from the wild-type control.

47 PV, 23 ET, and 8 PMF patients were reported *JAK2* V617F mutation positive after the TaqMan qPCR, which is one hundred percent in accordance with the result from snapback primer assay. The *MPL* TaqMan system identified no more *MPL* mutation in the 120 Ph-negative MPNs, as compared with the snapback primer assay. However, 1 PMF sample (J247) identified as *MPL* W515L positive by snapback assay showed a weak-positive curve with *C*_*t*_ = 38.623 in the W515L channel (Figure 6).

Results from the snapback primer system were further verified with Sanger sequencing. Ten *JAK2* V617F, 3 *MPL* W515L, 2 *MPL* W515K, and 10 *JAK2* V617F- and *MPL* W515L/K-free DNA samples were selected and subjected to the bidirectional sequencing. Four discordant results were reported, which were all negative in the sequencing but positive in the snapback primer system, 2 for *JAK2* V617F, 1 for *MPL* W515L (J247), and 1 for *MPL* W515K. After T-A cloning, the subclone of these 4 samples was again subjected to sequencing and turned out to harbor the mutant alleles, and supporting the snapback primer system enjoyed a higher sensitivity over the commonly used sequencing method.

## 4. Discussion

The last decade witnessed great strides in the development of molecular pathology of myeloproliferative malignancies. Soon after the discovery of mutations in *JAK2* and *MPL* in the middle 2000s, these molecular abnormalities have now become one of the most important diagnostic markers for the Ph-negative MPNs [19–21]. Moreover, the first FDA-approved JAK inhibitor Jakfi (Ruxolitinib) also changed our point of view on the clinical management of myelofibrosis [22, 23]. All these progresses in understanding, diagnosis, and treatment of disease mightily intensified the clinical demands for the accurate identification of these mutations.

At present, sequencing, ARMS, and fluorescent probe-based qPCR still remain the most available approaches for the detection of these genetic alternations. However, both the ARMS and sequencing technology are technically challenging and time/labor consuming. Besides, genetic analyzer is also rather expensive for the small-scale laboratories to afford. ARMS qPCR is a powerful tool for rare allele mutation detecting, because this system minimalized the time/labor cost of each test [24]. However, this SYBR dye base method still costs the false positive results that are introduced by the nonspecific amplification of ARMS. Several commercial assay kits, such as the Ipsogen MutaScreen, have been introduced for the sensitive semiquantification of *JAK2* V617F and common *MPL* mutations utilizing the minor grove binder (MGB) probe. However, the additional rare mutations in *JAK2* exon 14 and *MPL* exon 10, such as *JAK2* c.1860C>A (D620E) [25], c.1848T>C/1849G>T (C616C/V617F) [26], c.1849G>T/1853G>T (V617/C618F) [12], c.1849G>A (V617I) [27], *MPL* c.1543T>C (W515R) [28], and *MPL* c.1543T>G/1544G>C (W515A) [29], could lead to the failure of mutation detecting in the allele specific amplification or fluorescent hydrolysis probe method and bring out false-negative results.

The Wittwer Lab innovated snapback primer genotyping for single nucleotide polymorphism (SNP) in 2008 [30] and then modified it for the rare allele enrichment and detection in 2010 [13]. This simple and inexpensive snapback primer system integrates the PCR-derived rare-allele mutation enrichment and the melting curve analysis-derived mutation detection into a closed-tube reaction. The stem-loop hairpin structure of the single-strand DNA amplicon facilitates the selective amplification of the mutant allele, providing the snapback primer system with the favorable sensitivity to identify the trace amount of mutations with as low as 0.01–1% mutation load. Moreover, since the test result is evaluated with melting curve analysis, the snapback primer system can precisely segregate almost all the snapback probe-flanked small sequence alternations, such as SNP, insert, deletion, and even tandem repeats [31], thus minimizing the risk of false negative results induced by the rare mutation. Moreover, the unique melting temperature of each PCR product in melting curve analysis also improves the detection specificity and eliminates underlying false positive results.

In this study, we developed a robust multiplex snapback primer assay to detect *JAK2* V617F and *MPL* W515L/K mutations simultaneously. This multiplex system comprises two pairs of primers, each designed for the amplification of stem-loop hairpin flanking the* JAK2* and *MPL* mutation, respectively.

For the detection of mutations in the Ph-negative MPNs, especially the *MPL* W515L/K, extremely high sensitivity is necessary, since this group of diseases is characterized with polyclonal events [32]. Previous studies reported a variety of ingeniously designed assay methodologies for the sensitive detection of *JAK2* 617F and *MPL* W515L/K mutations, with the analytical sensitivity around 1–5% mutation load [4, 16, 33–35]. Our snapback primer system, with a robust reproducibility, can also discriminate the *JAK2* V617F and *MPL* W515L/K mutations simultaneously down to the 0.1% level.

The mutation detecting performance of the snapback primer assay was validated with 120 peripheral blood DNA from MPNs patients, in comparison with the electrophoresis based ARMS method and TaqMan qPCR. The test results were further verified with T-A cloning sequencing. Results of snapback primer assay and ARMS method were totally identified, and the new method also detected one indeterminable weak-positive *MPL* W515L mutation in a PMF patient, indicating a favorable detection sensitivity of the snapback primer assay.

Our multiplex system condenses the genetic test strategy for classical Ph-negative MPNs. According to the molecular diagnostic pipeline of disease, *JAK2* V617F is the initial test of all the suspicious PV, ET, and PMF patients [36]. For the patients with a wild-type *JAK2* exon 14, further *MPL* W515L/K mutation identification should be performed if the patients are suspicious for ET/PMF. Here, we combined the *JAK2* V617F and *MPL* W515L/K mutation detection with a multiplex system, which reduced the total processing time of molecular diagnosis of MPNs.

There are additional benefits of this system. Since the melting curve analysis strategy combines the fluorescent signal with the melting temperature of the PCR products, it provides our method with a favorable analytical specificity [37]. Besides, this closed-tube assay decreases the labor cost due to the simplified post-PCR analysis and thus reduces the risk of cross contamination.

Of note, we observed the coexistence of *JAK2* V617F and *MPL* W515K mutations in a clinical sample of ET patient. Although previous studies from Pardanani et al. [7] and Pietra et al. [38] reported this rare concurrent mutations of *JAK2* V617F and *MPL* W515L in ET patients, we could not exclude the possibility of plasmid/sample contamination. Although we did not detect any other mutations in this study except the *JAK2* V617F and *MPL* W515L/K, this snapback primer system might have the ability to identify other *JAK2* exon 14 and *MPL* exon 10 genetic alternations within the snapback probe covered region theoretically.

However, our system still has room for improvement. Firstly, the entire method was developed and validated on a high-resolution melting platform. The practicability of this system on other classical real-time instruments still needs to be further tested, although our previous work has reported the application of oligonucleotide probe melting analysis on the conventional devices [39]. Furthermore, the rapid PCR strategy for snapback primer assay is highly dependent on the air flowing PCR instrument [40]. Our preliminary data suggested that the quality of PCR product from a hot-lid thermal cycler was insufficient for the subsequent melting curve analysis.

In summary, we developed a novel labor-saving snapback primer system for simultaneous detection of *JAK2* V617F and *MPL* W515L/K mutations by melting curve analysis. With a favorable sensitivity and reproducibility, this closed tube system might be applied to the molecular diagnosis of Ph-negative MPNs.

## Figures and Tables

**Table 1:** Primers used for snapback primer assay, amplification refractory mutation system, TaqMan  probe qPCR, and Sanger sequencing.

	Primer	Sequence (5′-3′) and fluorescent label
Snapback	*JAK2* V617F snapback	*GG * GAGTATGTgTCTGTGGAGACTGACACCTAGCTGTGATCCTG*
	*JAK2* V617F limiting	TGAAGCAGCAAGTATGATGAG
	*MPL* W515L/K snapback	*AC * CTGCTGAGGtggCAGTTTCCTGGGGTCACAGAGCGAACCAA*
	*MPL* W515L/K limiting	AGCCTGGATCTCCTTGGTGAC

ARMS	*JAK2* V617F outer forward	TCCTCAGAACGTTGATGGCAG
	*JAK2* V617F outer reverse	ATTGCTTTCCTTTTTCACAAGAT
	*JAK2* V617F specific wild-type	GCTTTGGTTTTAAATTATGGAGTATATG
	*JAK2* V617F specific mutant	GTTTTACTTACTCTCGTCTCCACAAAA
	*MPL* W515L/K outer forward	GCCTGGATCTCCTTGGTGAC
	*MPL* W515L/K outer reverse	GAGGTGACGTGCAGGAAGTG
	*MPL* W515L/K specific wild-type	CTGTAGTGTGCAGGAAACTGTCA
	*MPL* W515L specific	GCCTGCTGCTGCTGAGATT
	*MPL* W515K specific	GCCTGCTGCTGCTGAGTAA

TaqMan	*JAK2* forward primer	AAGCTTTCTCACAAGCATTTGGTTT
	*JAK2* reverse primer	AGAAAGGCATTAGAAAGCCTGTAGTT
	*JAK2* wild-type probe	VIC-TCTCCACAGAcACATAC-BHQ1
	*JAK2* V617F probe	FAM-TCCACAGAaACATAC-BHQ1
	*MPL* forward primer	TAGCCTGGATCTCCTTGGTG
	*MPL* reverse primer	ACAGAGCGAACCAAGAATGC
	*MPL* wild-type probe	FAM-CTGCTGAGGtggCAGTTTC-BHQ1
	*MPL* W515L probe	FAM-CTGCTGAGGttgCAGTTTC-BHQ1
	*MPL* W515K probe	FAM-TGCTGCTGAGGaagCAGTTTCC-BHQ1

Sequencing	*JAK2* V617F seq. forward	CAAAGCACATTGTATCCTCA
	*JAK2* V617F seq. reverse	AGTCCTACAGTGTTTTCAGT
	*MPL*W515L/K seq. forward	AGCCTGGATCTCCTTGGTGACCG
	*MPL*W515L/K seq. reverse	TCACAGAGCGAACCAAGAATGCC

*For the snapback primer and TaqMan probe sequences, the italic uppercases stand for the 5′-mismatch of snapback probe; the snapback probe regions are underlined; the uppercases indicate the annealing primer; and the lowercases indicate mutation sites.
